# Hindguts of *Kyphosus sydneyanus* harbor phylogenetically and genomically distinct *Alistipes* capable of degrading algal polysaccharides and diazotrophy

**DOI:** 10.1128/msystems.01007-24

**Published:** 2024-12-23

**Authors:** Cesar T. Facimoto, Kendall D. Clements, W. Lindsey White, Kim M. Handley

**Affiliations:** 1 School of Biological Sciences, The University of Auckland, Auckland, New Zealand; 2 Department of Environmental Science, Auckland University of Technology, Auckland, New Zealand; University of Technology Sydney, Glebe, New South Wales, Australia

**Keywords:** *Alistipes*, fish gut microbiome, nitrogen, cobalamin, CAZyme, macroalgae

## Abstract

The genus *Alistipes* (*Bacteroidota*) is most often associated with human clinical samples and livestock. However, *Alistipes* are also prevalent in the hindgut of the marine herbivorous fish *Kyphosus sydneyanus* (Silver Drummer), and analysis of their carbohydrate-active enzyme (CAZyme) encoding gene repertoires suggests *Alistipes* degrade macroalgal biomass to support fish nutrition. To further explore host-associated traits unique to *K. sydneyanus*-derived *Alistipes*, we compared 445 high-quality genomes of *Alistipes* available in public databases (e.g., human and ruminant associated) with 99 metagenome-assembled genomes (MAGs) from the *K. sydneyanus* gut. Analyses showed that *Alistipes* from *K. sydneyanus* are phylogenetically distinct from other hosts and comprise 26 species based on genomic average nucleotide identity (ANI) analyses. Ruminant- and fish-derived *Alistipes* had significantly smaller genomes than human-derived strains, and lower GC contents, possibly reflecting a symbiotic relationship with their hosts. The fish-derived *Alistipes* were further delineated by their genetic capacity to fix nitrogen, biosynthesize cobalamin (vitamin B12), and utilize marine polysaccharides (e.g., alginate and carrageenan). The distribution of CAZymes encoded by *Alistipes* from *K. sydneyanus* was not phylogenetically conserved. Distinct CAZyme gene compositions were observed between closely related species. Conversely, CAZyme gene clusters (operons) targeting the same substrates were found across diverse species. Nonetheless, transcriptional data suggest that closely related *Alistipes* target specific groups of substrates within the fish hindgut. Results highlight host-specific adaptations among *Alistipes* in the fish hindgut that likely contribute to *K. sydneyanus* digesting their seaweed diet, and diverse and redundant carbohydrate-degrading capabilities across these *Alistipes* species.

**IMPORTANCE:**

Despite numerous reports of the *Alistipes* genus in humans and ruminants, its diversity and function remain understudied, and there is no clear consensus on whether it positively or negatively impacts host health. Given the symbiotic role of gut communities in the *Kyphosus sydneyanus* hindgut, where *Alistipes* are prevalent, and the diversity of carbohydrate-active enzymes (CAZymes) encoded that likely contribute to the breakdown of important substrates in the host diet, it is likely that this genus provides essential services to the fish host. Therefore, considering its metabolism in various contexts and hosts is crucial for understanding the ecology of the genus. Our study highlights the distinct genetic traits of *Alistipes* based on host association, and the potential of fish-associated *Alistipes* to transform macroalgae biomass into nutraceuticals (alginate oligosaccharides, β-glucans, sulfated galactans, and sulfated fucans).

## INTRODUCTION

*Alistipes* is a genus in the *Bacteroidota* (family *Rikenellaceae*). Members of the *Alistipes* are most often reported from human clinical samples (e.g., feces, appendix tissue, urine, abscesses) and livestock microbiomes (e.g., cow ruminal fluid and pig gut contents) (1–3). There is no clear consensus on whether *Alistipes* have a positive or negative impact on human health (1), although investigations of human and ruminant gut communities have indicated the presence of mucin-degrading enzymes in some *Alistipes* genomes (4, 5). In livestock, such as beef and dairy cattle, as well as broiler and pig production, *Alistipes* have also been attributed a role as growth enhancers due to their implied ability to degrade fiber and produce short-chain fatty acids (SCFAs) (1, 2, 6, 7). However, the majority of studies linking *Alistipes* with fiber degradation are primarily based on changes in the relative abundance of *Alistipes* alongside changes in diet, such as inclusion of plant-derived polysaccharides (e.g., cellulose) in ruminant and broiler chicken diets (2, 6, 7). Moreover, due to the varied hosts colonized by this genus and its high species diversity (1), it is likely that the ecological role of *Alistipes* extends beyond plant fiber utilization.

Studies of the herbivorous marine hindgut fermenter *Kyphosus sydneyanus* or Silver Drummer (family Kyphosidae) likewise suggest a relationship between *Alistipes* and dietary fiber utilization. These studies have indicated high relative abundance of *Alistipes* in distal sections of the hindguts of *K. sydneyanus* (8, 9), where carbohydrase activity and the fermentation products of dietary macroalgae (SCFAs) are also present at high levels (10, 11). *K. sydneyanus* are thought to be efficient marine polysaccharide degraders. However, digestion of refractory algal substrates likely depends on the enzymatic activity of the hindgut microbiome (9–11). Thus, *Alistipes* recovered from the *K. sydneyanus* gut are likely to be unique in terms of both carbohydrate-degradation capabilities and phylogeny.

The composition of dietary fiber consumed by *K. sydneyanus* differs considerably from most of the studied *Alistipes* hosts investigated to date. These other hosts are less likely to access polysaccharides such as alginate, laminarin, and carrageenan (predominantly found in brown and red algae), which are important components of the *K. sydneyanus* diet (12). In particular, the brown algal species *Ecklonia radiata* and the red algal species *Gigartina macrocarpa* are commonly found to dominate the stomach contents (15%–50% and 5%–30% of stomach content, respectively) of this fish species (12). Moreover, micro- and macroalgae, which comprise a significant fraction of marine carbohydrate biomass, display a distinct biochemical structure compared to terrestrial carbohydrates, particularly due to their sulfation (13). The structural modifications in these polysaccharides (e.g., sulfate decorations) confer resistance to various environmental stresses, as well as making them recalcitrant to enzymatic degradation (13).

Previous work from our group demonstrated substantial differences in taxonomic composition regarding taxa abundance and presence between the gut microbiota of *K. sydneyanus* and communities associated with its primary dietary alga *E. radiata* (8). Regardless of diet, location, and species, *Kyphosus* species accommodate *Alistipes* in their posterior gut, including *Kyphosus azureus* (14), *Kyphosus cinerascens* (15–17), *Kyphosus vaigiensis* (15–17), and *Kyphosus hawaiiensis* (16, 17). To date, most compositional studies of other herbivorous fishes, such as surgeonfishes (18, 19), parrotfishes (18, 19), rabbitfishes (18), marblefish (20), and butterfish (20), indicate that *Bacteroidota* (and consequently *Alistipes*) comprise minimum levels of relative abundance (<10%). Altogether, results indicate that *Alistipes* are likely to be specifically adapted to the anaerobic hindgut environment of *K. sydneyanus* and its dietary landscape. *Alistipes* may also be important in other herbivorous fish species. However, further work is necessary to address inconsistencies in methods for microbiota composition analysis and sampling of the fish gut, to gain a comprehensive view of *Bacteroidota* and *Alistipes* presence in the guts of various herbivorous fishes.

Our recent analysis of metagenome-assembled genomes (MAGs) recovered from the *K. sydneyanus* hindgut community showed that *Alistipes* from this fish indeed harbor an array of genes predicted to degrade macroalgal polysaccharides (21), including alginate, laminarin, fucose-containing sulfated polysaccharides (FCSP), and carrageenan, which comprise the main polysaccharides present in the fish diet (12). Here, we determined whether these metabolic capabilities are unique to the *Alistipes* in *K. sydneyanus* and investigated the extent of potential host-associated adaptations among *Alistipes*. We compared the genomic features and predicted the metabolic capabilities of *Alistipes* species obtained from both terrestrial hosts and *K. sydneyanus*. For this analysis, we included 59 MAGs that we previously generated from the hindguts of four *K. sydneyanus* individuals (21), and another 40 that we generated from an additional six individuals as part of this study. We also included 455 isolate genomes or MAGs of *Alistipes* from humans (*n* = 204), ruminants (*n* = 174), and other hosts/environments (*n* = 77). Results highlight the phylogenetic, genomic, and metabolic distinctiveness of *Alistipes* from the fish host, including an encoded composition of carbohydrate-active enzymes (CAZymes) that differs substantially from that of other known *Alistipes*, reflecting distinct glycan utilization capabilities, as well as nutrient and vitamin metabolisms that could compensate for deficiencies in host diet.

## RESULTS AND DISCUSSION

### *Alistipes* genomes recovered from fish and ruminants exhibit stronger host-associated adaptations

**Fig 1:**
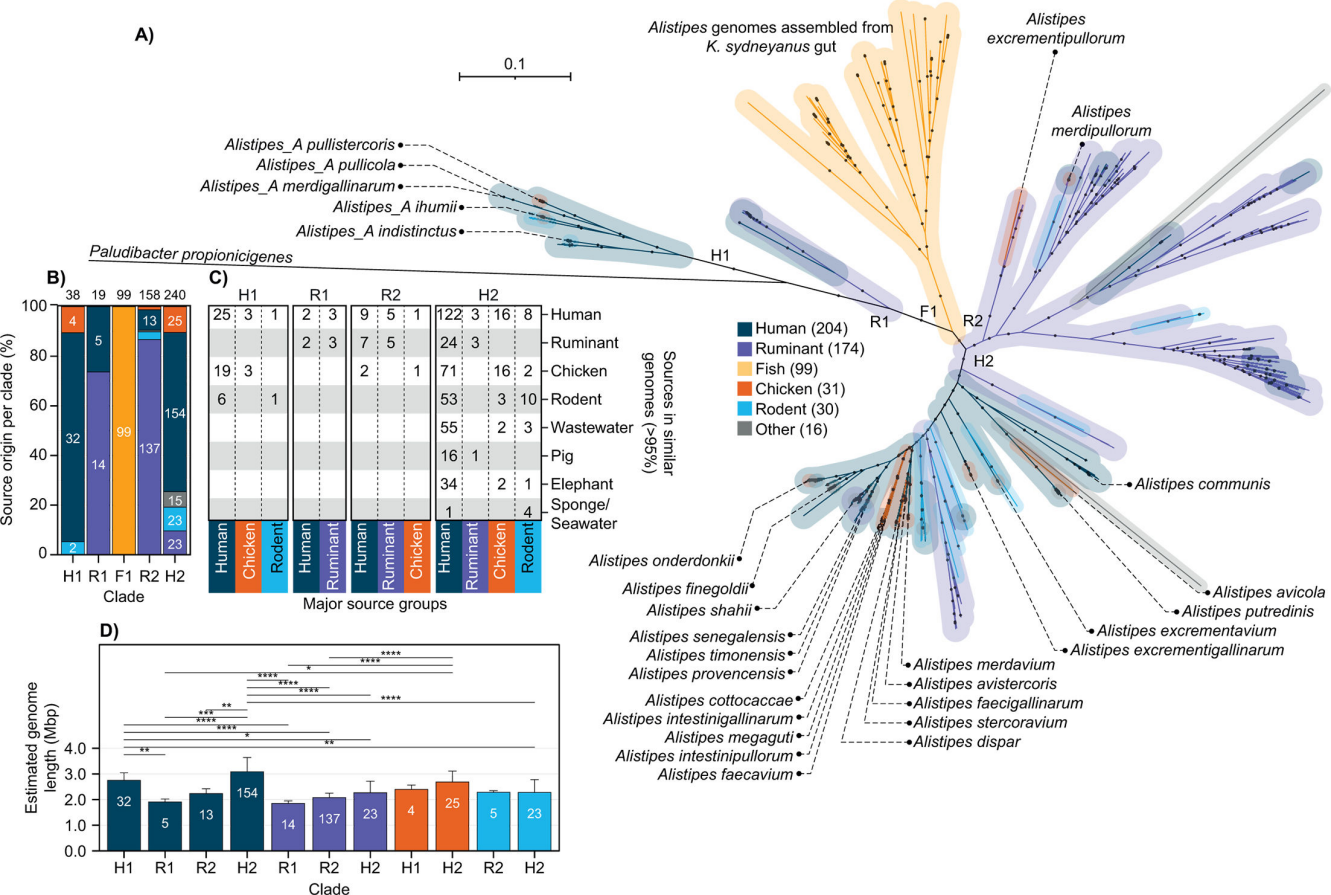
Tree with all the high-quality genomes used in this study (Table S3). (**A**) Tree branches are colored according to the source association that each genome was recovered. The alignment was generated by GTDB-Tk, and the tree was generated by FastTree using 1,000 bootstraps. Labels denote characterized species. The number of genomes in each source category is shown in the legend. The colored transparent background shading highlights group branches. Human, ruminant, and fish background shading is double the thickness of other groups. Black dots indicate bootstraps above 70%. Alphanumeric labels close to internal nodes represent major clades assignments. (**B**) Stacked bar plot showing the source origin of genomes in each clade. Numbers at the top of each bar indicate the total number of genomes in a clade. White labels within bars denote the number of genomes associated with source groups comprising more than 5% of the clade composition. (**C**) Number of genomes per clade that share >95% ANI (Table S3). (**D**) Mean estimated genome length across clades and source groups (only major source groups with at least three genomes in multiple clades are shown).

A total of 554 genomes (or MAGs) from multiple *Alistipes* species and environmental/host sources were compared to investigate the influence of different source associations, especially *Kyphosus sydneyanus* versus terrestrial hosts, on the phylogeny and genetic traits of *Alistipes* (Fig. 1B; Table S3). Most of the available reference genomes sourced from GenBank represented species from human samples (*n* = 204) followed by ruminants (*n* = 174), with a smaller number derived from chickens, rodents, and various other sources (Fig. 1; Table S3). The *Alistipes* from the *K. sydneyanus* hindgut (from longitudinal hindgut samples of 10 individuals, *n* = 99) formed a distinct phylogenetic clade (F1) with 100% bootstrap support (Fig. 1A). The *K. sydneyanus*-derived *Alistipes* genomes shared <95% average nucleotide identity (ANI) with each other and other *Alistipes* (Table S2). Ruminant-derived *Alistipes* were largely, albeit not exclusively, associated with a single clade R2 with 99.8% bootstrap support (137/158 genomes in clade R2, Fig. 1B). A small number of these ruminant genomes in R2 (5/137 ruminant-associated genomes in clade R2) shared >95% ANI with human-derived *Alistipes* (Fig. 1C; Table S3). In contrast, human-associated *Alistipes* were phylogenetically diverse. Most clustered within two clades (32 in clade H1 and 154 in clade H2, Fig. 1B), but 147 of these human-derived genomes (25 in H1 and 122 in H2, Table S3) shared >95% ANI with *Alistipes* from various terrestrial animal sources, wastewater, and marine environments (Fig. 1C). The phylogenetic similarity of some human- and animal-derived *Alistipes* may reflect the close physical association between humans and these other sources (mostly domesticated animals) (Fig. 1C). Results therefore highlight both the phylogenetic diversity of *Alistipes* across the different hosts, including many undescribed lineages from animal hosts, and the high host-specificity of *K. sydneyanus-* and ruminant-associated *Alistipes* (Fig. 1A).

**Fig 2:**
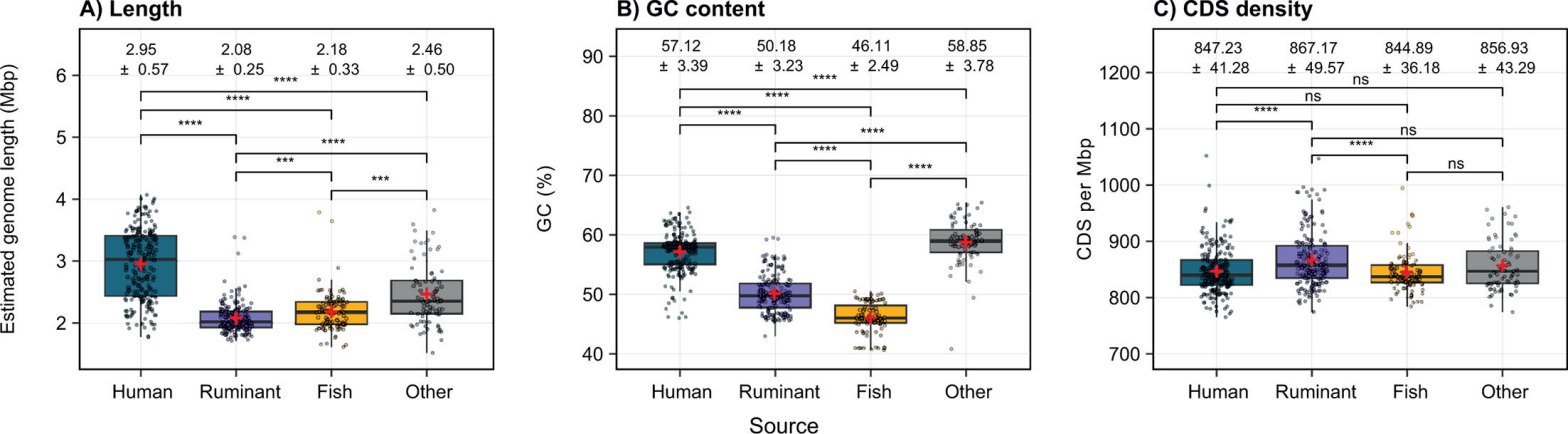
Genomic features of *Alistipes* genomes recovered from different sources. (**A–C**) Box plots show estimated genome length (corrected for completeness and contamination estimated by CheckM), GC content, and protein coding sequences (CDS) per megabase pair (Mbp) of *Alistipes* genomes across source groups. Points are colored by group and denote the observed values per genome. The boxes and central line represent the interquartile range and median across genomes per source group, and the whiskers represent the minimum and maximum values within 1.5 times the interquartile range. Upper brackets indicate the significance of pairwise groups. Mean values are indicated by red crosses, and standard deviation values are displayed at the top of the plots.

We compared the genomic features of *Alistipes* based on host or source (human, ruminant, *K. sydneyanus*, or other). These source-defined groups displayed significant differences in genomic features, such as genome length and GC content across all groups (Fig. 2). We found that the estimated genome sizes of *Alistipes* associated with *K. sydneyanus* (2.18 Mbp ± 0.33 standard deviation [SD]) and ruminants (2.08 Mbp ± 0.25 SD) were significantly lower on average than those associated with humans (2.95 Mbp ± 0.57 SD), which were 1.35–1.42 times larger on average, and those associated with *K. sydneyanus* and ruminants had the narrowest genome size distributions (Fig. 2A). The smaller size of *Alistipes* genomes from *K. sydneyanus* and ruminant hosts was not due to genome incompleteness. Most *K. sydneyanus*-derived (84.8%) and human-derived (80.4%) genomes included in the data set were estimated to be ≥95% complete (Fig. S1D). As such, estimated genome completeness and lengths were poorly correlated, and the weak correlation was driven largely by lower completeness of a subset of ruminant-derived genomes and genomes from other sources (Fig. S1A). When considering only genomes from ruminants with >95% completeness, the average size estimated for was 2.11 ± 0.29 SD, hence still appreciably smaller than those from humans. Given the mix of isolate and environmental genomes in the data set, we also considered the effect of assembly fragmentation (based on N50s) on estimated genome lengths (Fig. S1). Most genomes were similarly fragmented (*n* = 530) with N50/estimated genome length ratios of <0.5 (Table S3), whereas few genomes sourced from humans (*n* = 23) and rodents (*n* = 1) were represented by highly contiguous assemblies with N50/estimated genome length ratios >0.5 (and which also had >97% completeness). We observed a weak positive correlation between N50/estimated genome length ratios and estimated genome length (Fig. S1E); however, no significant differences in estimated genome lengths were observed when comparing highly contiguous assemblies versus less contiguous assemblies within the same species (Fig. S1F).

We also assessed the effect of phylogeny on genome size of host source. Human-associated genomes in human-dominated clades H2 and H1 displayed significantly higher genome lengths than human-associated genomes in ruminant-dominated clades R1 and R2, ruminant-associated genomes in any clade, and rodent-associated genomes in clade H2 (Fig. 1D). In contrast, chicken-associated genomes in clade H2, many of which share close similarity with human-derived genomes (>95% ANI, Fig. 1C), also featured significantly larger genomes than those in ruminant-predominant clades (human associated and ruminant associated in R1 and R2, Fig. 1D). Taken together, these observations suggest that host affinity is congruent with phylogenetic relatedness and that both influence genome length.

As low GC content is a feature of bacteria with reduced genomes (22–24), we also compared genome GC contents. Lower GC percentages were observed in genomes recovered from *K. sydneyanus* and ruminants than from human and other source groups (Fig. 2B; Fig. S1B). GC content is constrained by both environment and phylogeny (22). Higher GC contents are also associated with larger genomes (22) and retention of DNA repair mechanisms preventing AT bias through cytosine deamination (22–24). Despite the smaller genome sizes and lower GC contents of *K. sydneyanus-* and ruminant-associated *Alistipes*, analysis of coding densities (CDS per megabase pair) indicated a broadly similar “packing” of genes across source groups (Fig. 2C; Fig. S1C). Human-associated genomes are also significantly more heterogeneous regarding their individual gene GC contents than any source group (Fig. S2A). While this could potentially reflect a greater fraction of horizontally transferred genes in human-associated *Alistipes*, this requires further study.

The smaller genome size of *K. sydneyanus* and ruminant *Alistipes* implies these organisms have a more limited metabolic capacity compared to larger genomes from human and other sources (25). Although we cannot rule out a symbiotic association between some *Alistipes* from human and other sources, the genome reduction in *K. sydneyanus-* and ruminant-derived *Alistipes* genomes analyzed here could be a consequence of symbiotic relationships with their respective hosts (23, 24). Small genome sizes (smaller than 2 Mbp) typically reflect genome reduction to minimize replication costs, which can occur via streamlining (23) or in host adaptation (24). These are impacted by distinct evolutionary processes. Streamlining results from the selection of minimal yet efficient genomic capacity (e.g., resource uptake versus biosynthesis specialization in oligotrophic environments) (23). Genome reduction associated with host adaptation is instead associated with genetic drift, inactivation, and deletion of genes that are only weakly beneficial (24). Symbiotic microbes also obtain products and other benefits from the host and eventually lose genes associated with unused functions not under selective pressure (23, 24). Therefore, both processes likely explain the small genome sizes of *K. sydneyanus-* and ruminant-adapted *Alistipes*.

### Amino acid biosynthesis, nitrogen assimilation, and carbohydrate utilization distinguish *Alistipes* metabolism in *K. sydneyanus*

**Fig 3:**
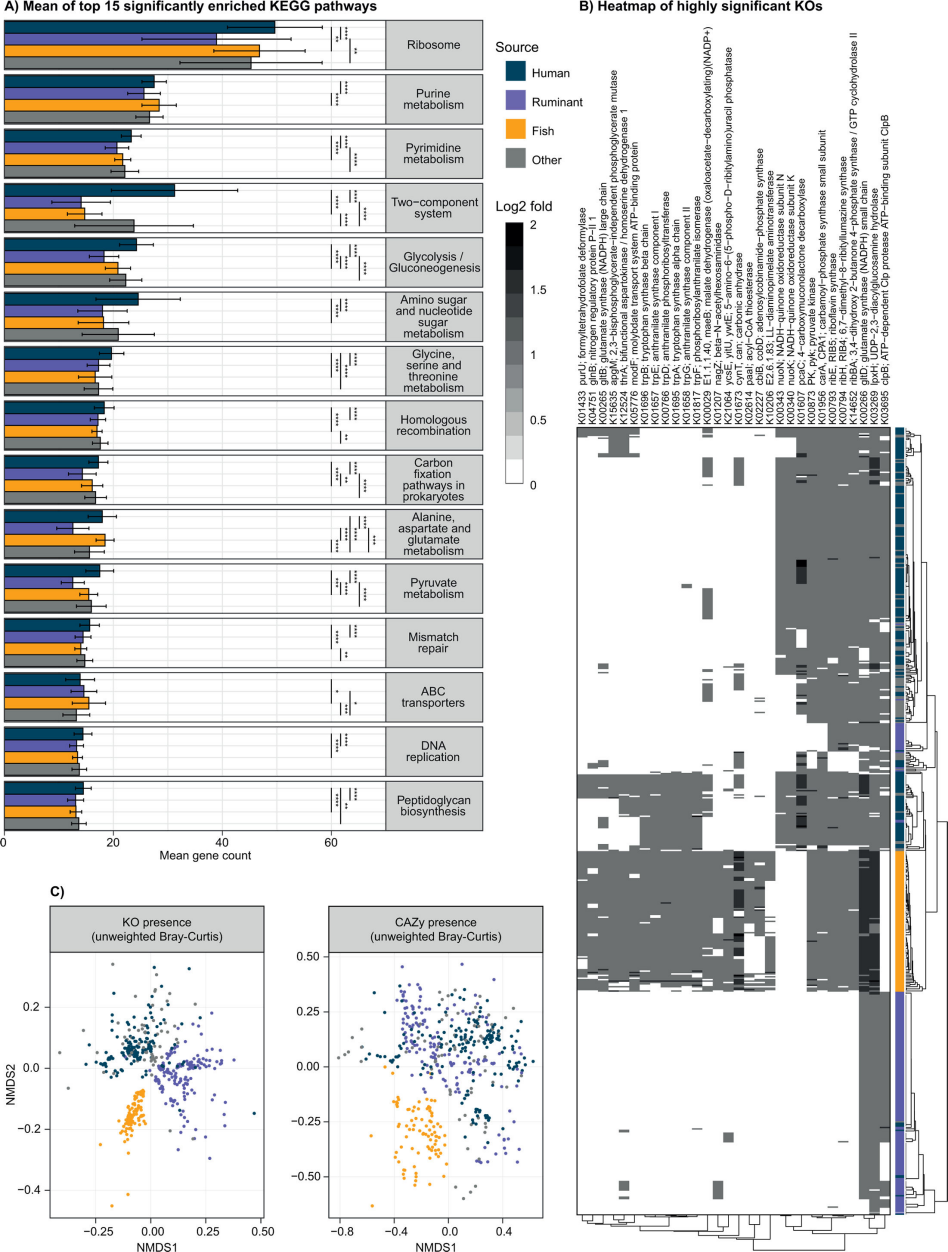
Metabolism exploration panel. (**A**) Bar plot of the 15 highest and significantly enriched (Kruskall-Wallis, *P* values < 0.05, and then Dunn test with Bonferroni correction, adjusted *P* values < 0.05) KEGG pathways. The category enrichment was estimated by the sum of KOs annotated within each genome and KEGG pathway. Unspecific KOs (assigned to multiple pathways) were counted in each respective pathway they belonged. Pathway categories are ranked by their average gene abundance per genome across source groups. (**B**) Heatmap of the KOs with the 30 lowest *P* values (Kruskal-Wallis, *P* values < 0.05, and Dunn test with Bonferroni correction, adjusted *P* values < 0.05) showing their gene abundance per genome across source groups. Box values are normalized to log2 fold with gray shading denoting the KO (x-axis) enrichment (dark boxes) per genome (y-axis). Dissimilarity was estimated using Bray-Curtis distances (vegan R package). The heatmap was generated using pheatmap. (**C**) Non-metric multidimensional scaling (NMDS) plot of composition of KOs and CAZy families . Unweighted dissimilarities were estimated using Bray-Curtis dissimilarities (vegan R package).

We identified the most significantly enriched functional KEGG pathways encoded by *Alistipes* genomes when comparing *Alistipes* from different hosts. Across most of these pathways, *Alistipes* from humans and “other” environments exhibited slightly higher gene abundances compared to *K. sydneyanus-* and ruminant-associated species (in 10 out of 15 pathways; Fig. 3A; Table S4). These modest pathway enrichments likely reflect expanded functional capacities derived from the longer genomes in these groups. There was a stark enrichment of genes in *Alistipes* from humans and other sources that are associated with the two-component system (TCS). This includes genes such as *fecR* (ferric citrate sensor) (26), *uhpC* (sugar phosphate sensor) (27), *kinB* (sensor histidine kinase associated with virulence modulation) (28), *kdpD* (potassium sensor histidine kinase) (29), *yesN* (carbohydrate utilization response regulator) (30), *zraR* (stress and starvation response regulator) (31), *ntrY* (nitrogen regulation sensor) (32), and *nreB* (oxygen/nitrate sensor) (33) (Fig. S2C; Table S5). The presence of these genes suggests enhanced encoding for machinery to sense environmental cues (e.g., related to osmolarity and resource availability) and regulate cellular processes such as virulence (34). TCS enrichment has been linked to free-living bacteria due to their complex lifestyle and their necessity to respond to multiple environmental stimuli (29).

Of the differentially enriched genes (KEGG orthologs, KOs) among host groups, those more enriched in human-associated *Alistipes* include KOs associated with oxidative stress or aerotolerance. In particular, results indicate the *nuoN* and *nuoK* genes from the NADH-ubiquinone oxidoreductase respiratory complex (Fig. 3B), and complete pathways for menaquinone biosynthesis were widespread in human-associated genomes (Fig. S3). The quinone-based respiratory chain facilitates the translocation of protons into the periplasm, conserving proton motive force (PMF) by using menaquinone as the electron acceptor and NADH as the electron donor (35). PMF supports ATP synthesis through F-type ATPases (36), which are encoded by genomes within clade H1 (Fig. S3). In contrast, reduced quinones act as electron donors for cytochrome *bd* oxidases, reducing oxygen into water (37). Accordingly, cytochrome *bd* oxidase genes (*cydA* and *cydB*) were widespread in human and subsets of ruminant *Alistipes* genomes from clade R2 (Fig. S2C), suggesting an aerotolerance mechanism in these bacteria. Transient exposure to oxygen (e.g., due to close proximity to the gut epithelium) can lead to interactions with flavin, metal centers, and quinone electron carriers, producing reactive oxygen species (ROS) such as superoxide and hydrogen peroxide (37, 38). ROS can disrupt the normal function of metalloenzymes and compromise DNA integrity (37, 38). Genes encoding superoxide dismutase (SOD), catalyzing the dismutation of superoxide radicals into hydrogen peroxide, and catalase (*katE*), converting hydrogen peroxide into water, were also widespread in *Alistipes* genomes in general (Fig. S2C). However, a select number of human-associated *Alistipes* also encoded peroxiredoxin (*ahpC*) genes, and a subset of genomes in ruminant-derived clade R2 encoded glutathione peroxidase (*gpx*) genes in addition to *katE* and SOD (Fig. S2C). Altogether, results indicate that some *Alistipes* species recovered from humans (and to a lesser extent ruminants) may exhibit greater flexibility in the environments they colonize compared to *Alistipes* from *K. sydneyanus*.

A clear delineation of *K. sydneyanus Alistipes* was observed when considering differentially enriched KOs (Fig. 3B). Overall, the genes enriched in *K. sydneyanus Alistipes* highlight the importance of amino acid biosynthesis in these taxa (Fig. 3B). The enrichment of amino acid biosynthetic genes within members of *K. sydneyanus* gut communities points to potential *de novo* protein biosynthesis contributing to host protein requirements (39, 40). This could be a response to the low protein content in brown macroalgae (e.g., protein content of *E. radiata* is 4.6%–7.8% of dry weight) (41) and comprises mainly non-essential amino acids such as glutamine, aspartate, glycine, and alanine (41, 42). Despite the substantial enrichment of amino acid biosynthetic genes in *K. sydneyanus*-associated *Alistipes*, human-associated clades H1 and part of H2, and certain genomes of ruminant-associated clade R2, also encoded complete biosynthetic pathways for threonine, leucine, isoleucine, and histidine (Fig. S3). In addition to protein production, these amino acids are central to branched-chain amino acid (BCAA) production (threonine, leucine, and isoleucine). BCAAs are involved in transcriptional regulation via leucine-responsive regulatory protein (Lrp), which is dependent on nutrient availability in Gram-negative bacteria (43), and in signal transduction via histidine protein kinases (44). In contrast, clade H2 and part of clade R2 lacked pathways for histidine biosynthesis but encoded pathways for histidine degradation (Fig. S3), implying that human- and ruminant-associated species scavenge histidine as a carbon, energy, and nitrogen source (32).

The tryptophan biosynthesis pathway was complete (e.g., *trpA*, *trpB*, *trpC*, *trpD*, *trpE*, *trpF*, and *trpG*) (45) only in certain *K. sydneyanus*-associated *Alistipes* and human-associated *Alistipes* from clade H1 (Fig. 3B; Fig. S3; Table S5), implying the capacity for tryptophan production in *Alistipes* by these hosts. Tryptophan is an essential amino acid for host protein synthesis and a precursor of metabolic regulators including serotonin, melatonin, and niacin (vitamin B3) (46). The glutamate synthase large subunit gene, *gltB*, was likewise almost exclusively present in *Alistipes* genomes from *K. sydneyanus*. These *Alistipes* possess both *gltB* and *gltD* genes. Both large (GltB) and small (GltD) subunits are required for the enzyme to be functional (47), and their presence indicates the capacity to control ammonia and glutamate/glutamine levels through the glutamine synthetase/glutamate synthase (GS/GOGAT) cycle (48) (Fig. 3B). The conversion of glutamine to glutamate through GltBD also provides nitrogen for the biosynthesis of tryptophan from chorismate (45). As most other *Alistipes* lacked the *gltB* gene, these other groups are unlikely to convert glutamine to glutamate but may synthesize glutamate from ammonia via the *gdhA* glutamate dehydrogenase gene (49) (Fig. S2C).

When considering all genes associated with nitrogen metabolism present, those associated with nitrogenase were almost uniquely present in a large group of *Alistipes* from *K. sydneyanus*. This included nitrogenase genes such as *nifH* (K02588), *nifD* (K02586), and *nifK* (K02591) (Fig. S3), which encode the nitrogenase molybdenum-iron alpha chain enzyme, and a *nifA* transcription activator (Fig. S2C). Diazotrophy in *K. sydneyanus* gut communities was demonstrated previously by our group, showing *nifH* gene expression and rates of nitrogen fixation comparable to termites (40). Together, these results suggest *Alistipes* may contribute to nitrogen assimilation in the *K. sydneyanus* gut (Fig. S3). This is further supported by the presence of other genes pertinent to diazotrophy, such as *modF*, which is associated with the uptake of molybdate (50) (a key cofactor for some nitrogenases), and the *glnB* nitrogen regulator, which controls the flow of nitrogen derived from nitrogen fixation (51) (Fig. 3B). Diazotrophic microbiota may be important for fish protein metabolism given the low levels of protein and nitrogen in the fish diet (40, 41).

The presence of genes involved in certain vitamin biosynthetic pathways is also characteristic of the *K. sydneyanus* group. These included *ribBA* (K14652), *ycsE* (K21064), *ribH* (K00794), *ribE* (K00793), and *ribF* (K11753) for riboflavin synthesis (Fig. S2), and *cobA* (K19221), *pduO* (K00798), *cobQ* (K02232), *cbiB* (K02227), *cobP* (K02231), *cobS*/*cobV* (K02233), and *cobU*/*cobT* (K00768) for cobalamin synthesis from cob(II)yrinate diamide (Fig. 3B; Fig. S3). Cobalamin (a cobamide) operates as a cofactor for central metabolic processes, such as carbon metabolism (52), biosynthesis of methionine (52) (an essential amino acid present in low levels in *K. sydneyanus* diet) (42), and deoxynucleotides (52). For example, *de novo* cobalamin (vitamin B12) biosynthesis is pivotal for the biosynthesis of methionine via methylcobalamin-dependent methionine synthase (52). Methionine is a precursor of cysteine (53) and a cofactor for methylmalonyl-CoA mutase, which coverts methylmalonyl-CoA into succinyl-CoA as part of propionate metabolism (52, 53). However, complete cobamide biosynthetic pathways are found in only 37% of bacteria and only 0.6% of *Bacteroidota* (52), suggesting prevalent interspecies cobamide dependence within microbial communities (52).

**Fig 4:**
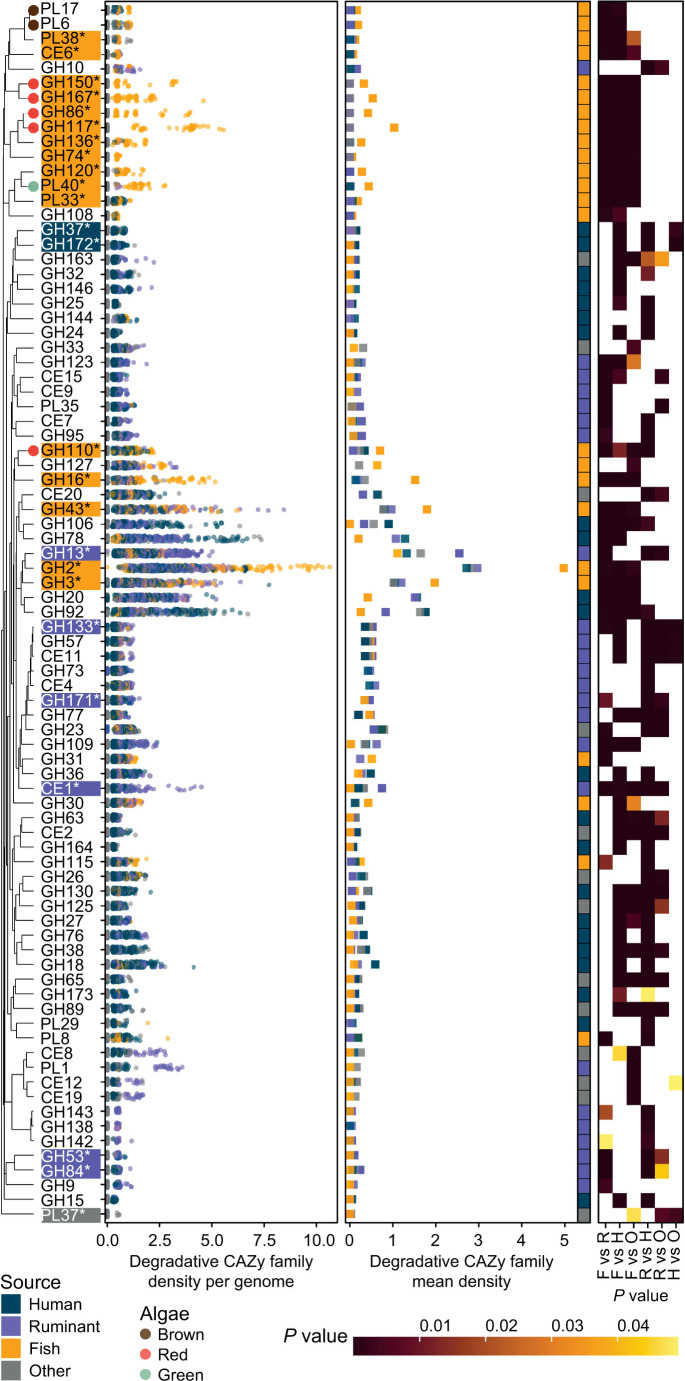
Degradative CAZy families are differentially dense across source groups. CAZy family densities show the number of gene copies for each family per megabase pair. Leftmost dendrogram shows the Bray-Curtis dissimilarity of degradative CAZy families (GH, PL, and CE) density across genomes. Dendrogram labels indicate each significantly dense CAZy family, and colored bubbles at the tips indicate families associated with algae degradation. CAZy families marked with an asterisk and background color indicate that the densest source group (background color) is significantly different from all groups. Panel immediately right of dendrogram displays the density (x-axis) of each CAZy family (y-axis) per genome (individual points) colored by source group. The following pane displays the mean CAZy family density (x-axis) in each source group represented by colored squares. Colored tiles on the right indicate the densest source group. Rightmost heatmap represents *P* values (Dunn test with Bonferroni correction, adjusted *P* values < 0.05) of pairwise comparisons (x-axis; F vs R, fish vs ruminant; F vs H, fish vs human; F vs O, fish vs other; R vs H, ruminant vs human; R vs O, ruminant vs other; H vs O, human vs other) for each CAZy family density across source groups. Significant comparisons are colored with yellow-to-brown shaded tiles (light yellow, low significance; dark brown, high significance). White tiles indicate non-significant comparisons.

The diet of *K. sydneyanus*, which comprises mainly brown and red algae (12), is distinct from that of other *Alistipes* hosts (13). Other *Alistipes* are thus unlikely to be specialized in the breakdown of polysaccharides such as alginate and laminarin from brown algae, or heavily sulfated polysaccharides, such as carrageenan, from red algae (12, 54, 55). Our previous analyses showed that *Alistipes* from the *K. sydneyanus* hindgut harbor an extensive array of CAZy families associated with the degradation of macroalgal polysaccharides (21). CAZy families consist of enzymes with similar structural folds, catalytic machinery, and reaction mechanisms (56). Despite shared structural and kinetic features, enzymes within the same CAZy family may exhibit a range of specificities (56, 57), implying a greater functional diversity beyond the family designation (56). Here, we identified similar frequencies of CAZyme genes for each host/source group, with an average of 38.40 genes/Mbp ± 11.11 SD for *K. sydneyanus*, 35.21 ± 11.15 SD for ruminants, and 36.22 ± 11.26 SD for humans (Fig. S2). However, *Alistipes* from *K. sydneyanus* displayed highly distinctive CAZyme family gene compositions (Fig. 3C), indicating a large difference in their genetic capacity for carbohydrate degradation compared to other *Alistipes*. The same results were obtained whether considering gene compositions based on presence/absence or abundance (Fig. 3C versus Fig. 4; Table S6). In contrast, the composition of CAZy families encoded by human- and ruminant-derived *Alistipes* is highly similar (Fig. 3C), although there were some differences in CAZy family gene densities between these source groups. For example, CAZy families associated with degradation of starch (amylases: GH13 and GH133), host glycan (hexosaminidases: GH171, and glucosaminidases: GH84), and lignocellulose (xylan esterases: CE1) were more frequent in the genomes from ruminants than any other source group (Fig. 4). In contrast, CAZymes from families GH37 (trehalases) and GH172 (fructofuranosidases) acting on glucose and fructose oligomers were more densely encoded in the human-derived *Alistipes* genomes than in any other group (Fig. 4).

We assessed the nature of encoded CAZyme family differences in the *K. sydneyanus Alistipes* clade and the capacity of other host/source groups to degrade major macroalgal substrates. This showed that algae-associated CAZyme genes enriched in, or unique to, *K. sydneyanus* comprised carrageenases, agarases, and galactosidases targeting red algae (GH150, GH167, GH86 families containing endo β-1,4 galactanases, and GH117 and GH110 containing α-galactosidases) targeting red algal polysaccharides, ulvan lyases (PL40) targeting green algae polysaccharides, and alginate lyases (PL6 and PL17) targeting brown algal polysaccharides (Fig. 4). Some of these CAZy families (PL17, PL6, PL40, and GH110) were also found in other source groups but at lower frequencies (Table S6). The presence of algae-associated CAZy families has been reported in human microbiomes (alginate: PL17, PL15, and PL6; agar: GH50, GH86, and GH117) (58, 59) and linked to human seaweed consumption. Similarly, ruminant microbiomes are also previously reported to carry algae-associated CAZy families such as PL40, PL6, and PL17 (5, 60). However, these are linked to the breakdown of host glycans (5), presumably due to their activity on glucuronic acid-containing polysaccharides. Nonetheless, CAZyme genes encoded by *Alistipes* from humans and ruminants contained far fewer genes associated with macroalgal degradation than those from *K. sydneyanus*.

### Numerous *Alistipes* species with distinct carbohydrate utilization potentials are harbored by the *K. sydneyanus* gut

**Fig 5:**
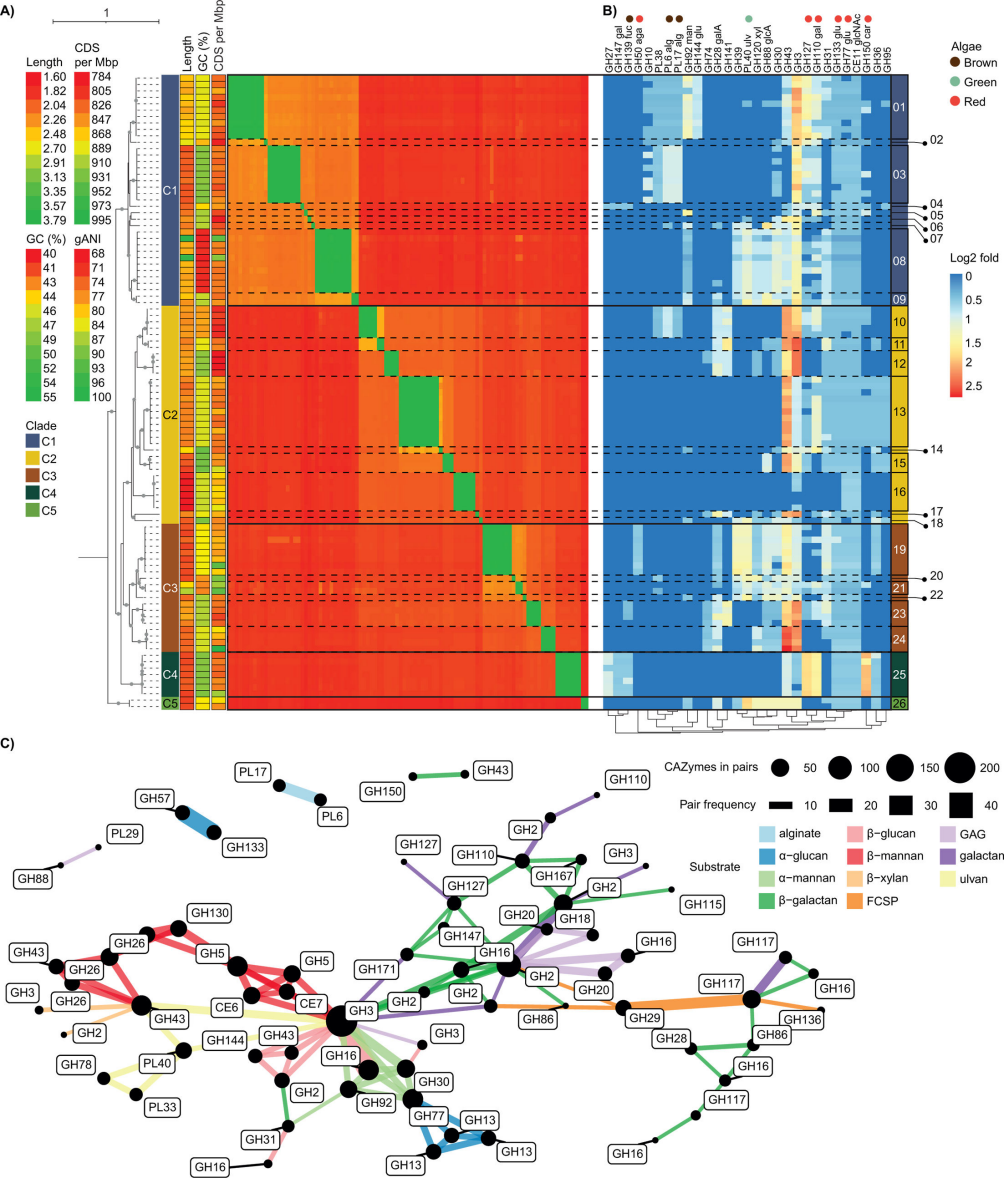
Phylogeny, genomic features, and differential carbohydrate capacity among *Alistipes* species from *K. sydneyanus* gut. (**A**) Phylogenetic tree of *Alistipes* genomes recovered from *K. sydneyanus* gut (*n* = 99); bootstraps shown as solid gray circles are above 70%. Colored labels immediately right of the tree indicate five major fish-associated phylogenetic clades. The following three heatmap columns indicate estimated genome length, GC content, and protein coding density per genome. The larger heatmap shows the genome pairwise ANI values indicating genome pairs with the highest ANI in green and the lowest in red. (**B**) Heatmap of differentially dense CAZymes across *Alistipes* clades. Differences were determined based on a Kruskal-Wallis test (*P* values < 0.05) followed by a Dunn test with Bonferroni correction (adjusted *P* values < 0.05). Values of each CAZyme are scaled by log2 and range from lower density (blue) to higher density (red). Dissimilarities for columns (CAZy family) were estimated using Bray-Curtis (vegan R package). Labels on the top indicate the CAZy family annotation followed by an abbreviation of their activity. Galactosidase, "gal"; fucosidase, "fuc"; agarase, "aga"; alginate lyase, "alg"; mannanase, "man"; glucanase, “glu”; galacturonidase, "galA"; ulvanase, "ulv"; xylanase, "xyl"; glucuronidase, "glcA"; N-acetyl glucosaminidase, “glcNAc”; and carrageenase, "car." Colored bubbles denote the potential activity of the CAZy family targeting macroalgae in the fish diet. Rightmost colored tiles indicate genome clade placement, and labels denote genomes corresponding to a species. (**C**) Network of CAZyme pairing within CAZyme gene clusters (CGCs). Edge width indicates the frequency a CAZyme pair occurred across CGCs. Black bubbles denote the summed frequency of a CAZy family orthogroup in any pair, and labels indicate the CAZy family. Colored edges are estimates of the substrates that each CAZyme association could act on (Table S10). Only pairs present in at least five CGCs are displayed to improve visualization.

GTDB-Tk taxonomically classifies prokaryotes based on relative evolutionary divergence and average nucleotide identity (61, 62). *Alistipes* MAGs from *K. sydneyanus* were unclassified at the species level (Fig. 1), indicating their distinctiveness. To further investigate diversity, we calculated pairwise ANI and alignment fractions (AFs) between *Alistipes* genomes from all sources to define species-level thresholds for the *K. sydneyanus Alistipes* (63) (Fig. S4). Based on AF 40% and ANI 96.5% thresholds, the *Alistipes* genomes from *K. sydneyanus* were nominally assigned to 26 species (Fig. 5A; Table S8). The boundaries for the species delineation estimated from ANI followed variations in genomic signatures such as GC content and estimated genome length (Fig. 5A). Phylogenetic clustering of these genomes based on 120 conserved single-copy bacterial genes (62) showed these newly inferred species clustered within five phylogenetic clades (Fig. 5A). Results thus indicate high intra-genus diversity among *Alistipes* within the *K. sydneyanus* gut.

To relate phylogenetic diversity to function, we measured the encoded CAZy family densities across *Alistipes* clades. Results show a high diversity of glycan utilization potential across clades (C1–C5) (Fig. 5B). Some encoded CAZy families were specific to a clade and were characteristic of members of certain species in those clades, including GH50 (β-agarase) in species 19 from C3, GH10 (β-xylanase and β-glucanase activity) in species 01, 02, and 03 in C1, and GH144 (β-glucanase) in species 01 from C1, indicating some level of niche specificity (Fig. 5B). However, many other CAZy families were more widely distributed. Genes encoding CAZy families predicted to act on substrates such as alginate (PL17 and PL6 in clades C1 and C2), FCSP (GH139 in clades C1, C3, and C4), mannan (GH92 in clades C1, C2, C3, and C5), ulvan (PL40 in clades C1, C2, C3, and C5), xylan (GH120 in clades C1, C2, C3, and C5), and carrageenan (GH150 in clades C1, C2, C3, and C4) were dispersed across genomes independent of phylogenetic clade placement (Fig. 5B). In addition, an assorted distribution of CAZyme genes was evident even among closely related species (e.g., genomes within the same clade), suggesting a poor correlation between trait presence and phylogeny. This varied distribution of CAZyme-encoding genes could be a result of horizontal gene transfer (HGT) among sympatric species within the *K. sydneyanus* gut. For example, host cohabitation is indicated to increase interspecies recombination up to sixfold in *Campylobacter* species from cattle (64). Closely related *Bacteroides ovatus* and *Bacteroides xylanisolvens* strains from terrestrial mammals exhibit extensive genomic diversification due to HGT (accessory genome = 82.5%, including a high proportion of genes involved in carbohydrate metabolism) (65).

### CAZyme gene clusters associated with distinct polysaccharide degradation pathways

CAZyme gene clusters (CGCs) are tightly linked clusters containing at least one CAZyme and at least one CGC signature gene that encodes for proteins, such as transporters (TCs), signal transduction proteins (STPs), or transcriptional factors (TFs) (66). These clusters are also termed polysaccharide utilization loci (PULs) and are often reported to confer an advantage in the breakdown of complex glycans in substrate-competitive environments by affording the orchestrated co-expression of complementary CAZymes (67, 68). The identification of CGCs and complementary CAZy families arranged in clusters can provide a powerful means to predict CAZy family activity and consequently bacterial “carbohydrate preference” (56). We previously showed *Alistipes* genomes were enriched in CGCs associated with macroalgal utilization relative to the wider fish hindgut community (21), which suggests they are specialist macroalgal degraders. Here, we expand on this finding to investigate CGC diversity among *K. sydneyanus*-derived *Alistipes* and their transcriptional activity. Results are based on analysis of a larger set of genomes (99 versus 20 previously), illustrate a large diversity of macroalgal substrate targets, and provide evidence for niche partitioning among *Alistipes* in the hindgut.

**Fig 6:**
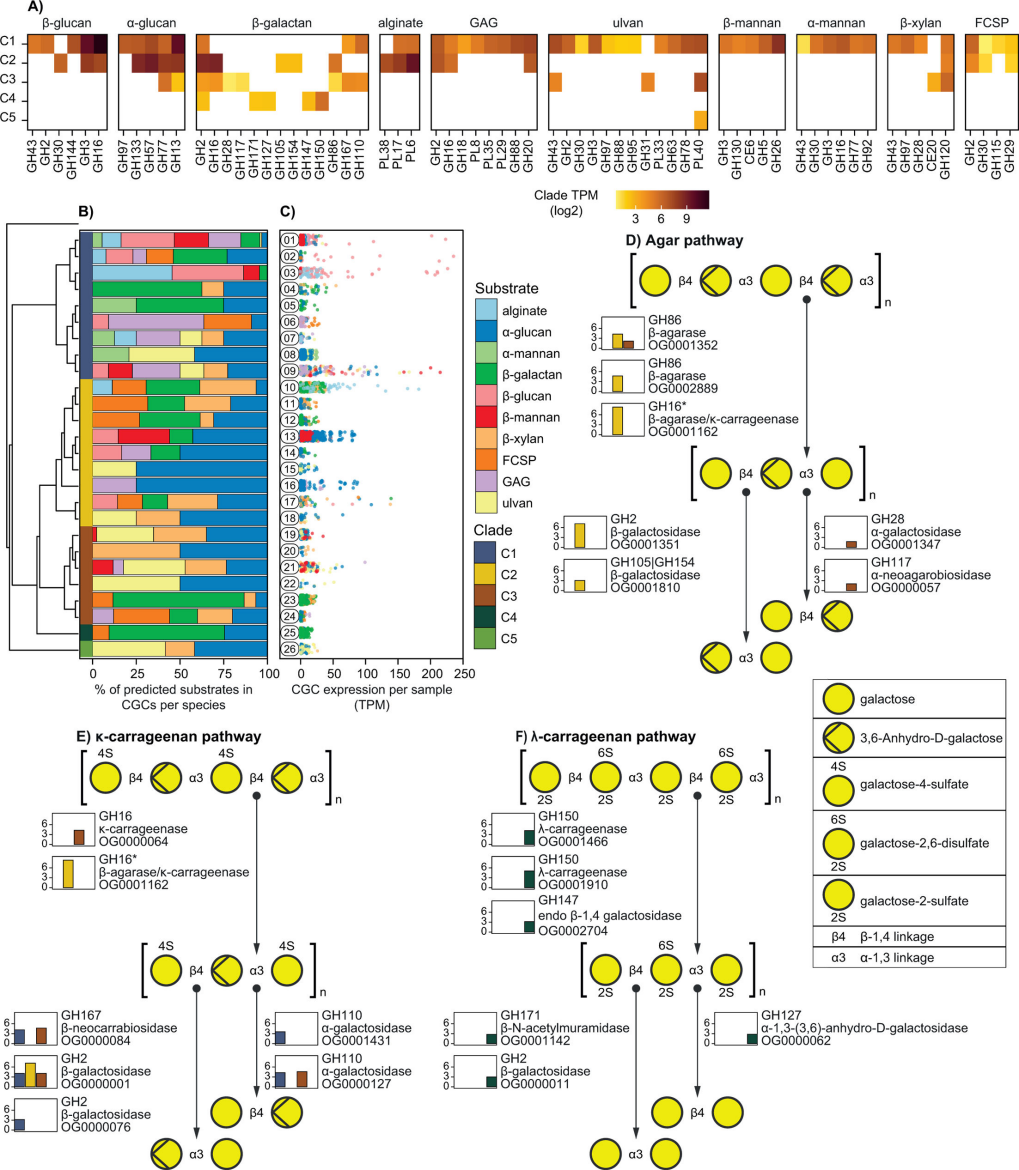
CGC abundance and expression in *Alistipes* from *K. sydneyanus*. (**A**) Heatmap of CAZymes by predicted substrate that were expressed in at least half (more than seven) of the samples. Expression values (TPM) are the sum of each CAZy family per clade per substrate-assigned CGC. They are scaled by log2 and range from not expressed (white) to highly expressed (dark yellow). Top labels indicate the substrate prediction of the CGCs. (**B**) Phylogenetic tree (left) showing the *Alistipes* species clustering. Colors at the tips of the tree indicate the clade assignment. Stacked bar plot shows the proportion of predicted CGCs in each species. (**C**) Dot plots display the expression in TPM of each CGC (sum of all genes within a CGC) in each sample and species. Rounded labels on the left denote the species ID, and dot colors represent the predicted substrate. (**D–F**) β-galactan degradation pathways based on CGC annotations, and gene expressed in more than seven samples. Bar plots represent CAZyme expression by CGCs associated with β-galactan degradation (values = log2-transformed sum of CAZy family and their orthogroup per clade) (bar colors as in B). Bar plots marked with an asterisk represent CAZymes associated with more than one type of galactan.

We found evidence for a total of 2,257 CGCs across all *K. sydneyanus*-derived *Alistipes* genomes (Table S11). A common feature of CGCs within these genomes was the pairing of CAZymes encoding for complementary activities to attack a substrate (Fig. 5C; Table S10). Such substrate-oriented CAZyme pairs included those associated with specialized families such as PL40 (ulvanases); GH26 or GH130 (β-mannanases); GH13, GH133, or GH57 (α-glucanases); PL17 or PL6 (alginate lyases); GH150 or GH167 (carrageenases); GH86 (agarases and porphyranases); GH92 (α-mannanases); and GH144 (β-1,2 glucanases) (Fig. 5C). Any other CAZyme paired with these within a CGC would contribute to the degradation of high molecular weight substrates present in the *K. sydneyanus* diet. Moreover, these associations suggest multiple pathways to attack certain substrates. This is observed in GH133 pairing with GH57, and GH77 pairing with GH13 CAZymes, whereby both pairs are predicted to attack α-glucans (e.g., starch or glycogen) (Fig. 5C). The diversity of degradation pathways encoded by the *Alistipes* CGCs (Fig. 6) could confer an ecological advantage at the populational level, as arrangements could facilitate efficient polysaccharide breakdown, or resource partitioning (e.g., utilizing different isoforms of a polysaccharide) (69).

To determine transcriptionally active CGCs among *Alistipes* in the hindguts of *K. sydneyanus*, we mapped RNA reads to MAGs and assessed their expression across phylogenetic clades. CGCs targeting glycans, such as alginate (*Alistipes* species 10 and 03) and β-glucan (species 01, 02, and 03) from brown algae, were among those expressed. CGCs targeting α-glucan (species 09, 13, and 16) and β-galactan (species 04, 05, 10, 12, 14, 23, and 25) from red algae were also expressed (Fig. 6B and C). Together, this indicates active degradation of major substrates (alginate, laminarin, and carrageenan) in the adult *K. sydneyanus* diet (12). Predicted CGCs targeting β-mannan that were expressed in *Alistipes* species 09, 19, and 21 were previously linked to the degradation of diatom biomass in the North Sea (70) and may indicate a role for diatoms in the *K. sydneyanus* diet, potentially as dissolved organic matter (71) or associated with kelp (72). The expression of CGC genes targeting the same substrate (e.g., β-galactan and α-glucan) across different clades within the same fish and gut section suggests the same substrates support the coexistence of phylogenetically distinct *Alistipes* (Fig. 6B and C; Fig. S5). Nonetheless, the summed expression of CGC elements (e.g., CAZyme, transporters, and regulators) displayed clade-specific patterns of transcription across samples for each substrate, suggesting an affinity of some clades toward specific substrates (Fig. S5). Therefore, despite shared traits across distinct phylogenetic groups (Fig. 6B), variations in CAZyme gene expression across clades (Fig. S5) likely support the coexistence of multiple species via niche partitioning enabled by highly complex substrate landscape in the *K. sydneyanus* gut (73, 74).

Of the five *Alistipes* clades identified from the *K. sydneyanus* hindguts, clade 1 exhibited the widest repertoire of CAZyme genes expressed compared to other groups (Fig. 6A), whereas clade 2 exhibited the largest effort toward CGC gene transcription (32.3%–99.8% of the total CGC expression per sample, Fig. S5), suggesting species in these clades play a greater role in carbohydrate degradation than other clades in *K. sydneyanus* digestion. The diversity of transcribed CAZy families associated with β-galactan utilization (Fig. 6A) further suggests different degradation pathways/enzymes across clades to degrade isoforms of this substrate (e.g., κ-carrageenases in clades C2 [GH16_13] and C3; β-agarases in clades C2 [GH86 and GH16_14] and C3 [GH86]; and λ-carrageenases in clade C4 [GH150]) (Fig. 6D through F; Table S12). Therefore, encoded endo-acting κ-carrageenase, β-agarase, and λ-carrageenase and their complementary exo-acting repertoire suggest distinct approaches to the degradation of κ-carrageenan by clades C1, C2, and C3, agar by clades C2 and C3, and λ-carrageenan by clade C4 (Fig. 6D through F). Altogether, these results indicate a diversified carbohydrate utilization capacity among *K. sydneyanus-*associated *Alistipes* species that likely confers competitive advantages depending on the substrate landscape (21, 75).

The genus *Alistipes* is a diverse taxonomic group spanning a variety of environments and hosts. Despite their widespread presence, their metabolism is relatively unexplored, particularly in the rumen, where *Alistipes* are often present. Our results suggest that *Alistipes* species in *K. sydneyanus* are adapted to their host. Furthermore, the *K. sydneyanus*-associated *Alistipes* were distinguished from other species due to their ability to fix nitrogen and biosynthesize cobalamin and tryptophan. Carbohydrate utilization, inferred from the CAZyme gene composition, differed across source groups and indicates *K. sydneyanus*-derived *Alistipes* are specialized toward marine glycans such as ulvan, alginate, and carrageenan, implying the evolution of CAZyme gene repertoires specific to substrates in the marine environment and host diet. *Alistipes* from the *K. sydneyanus* hindgut displayed distinct assortments of CAZymes and CGCs, independent of their phylogeny. Nonetheless, the diverse species within the *K. sydneyanus* gut are suggested to be functionally convergent, exhibiting complementarity with respect to glycan degradation or niche-partitioning of glycan resources at the population level via the utilization of multiple degradation pathways (e.g., β-galactan). The densely encoded biosynthetic and glycan utilization machinery identified in *Alistipes* from the *K. sydneyanus* gut indicates a pivotal role for this genus in converting marine polysaccharides into simpler substrates for other gut microorganisms and the fish host.

## MATERIALS AND METHODS

### Sample collection and sequencing

Six *K. sydneyanus* individuals were collected from waters around Great Barrier Island, Auckland, New Zealand, in January 2020. Fish collection was covered by approval 001636 from the University of Auckland Animal Ethics Committee. The fish were speared on snorkel and immediately dissected to separate their hindguts into three sections (III, IV, and V) (11). The stomach was designated as section I and the hindgut chamber as section V. The intervening gut was divided into equal lengths as II (immediately after the stomach), III, and IV (immediately before the hindgut sphincter) (Fig. S6). Gut contents from each gut section of each fish were homogenized, aliquoted into microtubes, and immediately stored in liquid nitrogen until storage in a −80°C freezer. Immediately prior to extraction, samples were thawed and centrifuged at 15,000 × *g* for 2 min, and the supernatant was removed. The leftover pellet was reconstituted using solution CD1 from the PowerSoil Pro kit (QIAGEN, Germantown, MD, USA), and DNA was extracted following the manufacturer instructions. DNA yields were measured using a Qubit 3.0 fluorometer with the dsDNA Quantification Assay Kit (Thermo Fisher Scientific, Waltham, MA, USA), and purity was determined using a NanoPhotometer (Implen, Munich, Germany). DNA was visually inspected using 0.8% agarose gel electrophoresis.

A total of 18 high-molecular DNA samples from sections III, IV, and V of the six fish were used for metagenomics. DNA libraries were prepared at the Otago Genomics Facility (University of Otago, Dunedin, New Zealand) with the Takara Thruplex DNA-Seq 96D kit (Takara Bio, Kusatsu, Shiga, Japan). Paired 125 bp reads were generated using the Illumina HiSeq V4 platform (Illumina, San Diego, CA, USA), yielding 442.4 Gbp of sequence (Table S1).

A subset of 13 samples (three section III, four section IV, and six section V) were used for metatranscriptomics based on detectable RNA of good quality. First, RNA was extracted, and DNA removed, with the Monarch Total RNA Miniprep Kit (New England BioLabs, Ipswich, MA, USA) following the manufacturer instructions. RNA yields were measured using Qubit and purity using a NanoPhotometer. DNA removal was confirmed by no amplification of the 16S rRNA gene in extracted versus positive control samples (76). RNA was purified using the RNA clean and concentrator-5 kit (Zymo Research, Irvine, CA, USA). RNA integrity was then evaluated using a 2100 Bioanalyzer and RNA Analysis kit (Agilent, Santa Clara, CA, USA), showing RNA Integrity Number (RIN) values from 5.5 to 7.6 for samples sequenced. An extra step of purification was performed using the AMPure XP kit (Beckman Coulter, Brea, CA, USA) to improve 260/230 purity ratios. cDNA libraries were prepared using the Zymo-Seq RiboFree Total RNA Library Kit (Zymo Research, Irvine, CA, USA) by Otago Genomics (Dunedin, New Zealand). Sequencing was performed using three Illumina NextSeq 2000 P3-200 flow cells, outputting a minimum of 110 Gb per sample of 2 × 100 bp paired-end (PE) reads (Table S1).

### Filtering, assembly, binning, and dereplication

Raw metagenomic reads were trimmed and adapters were removed using BBMap version 37.93 with the BBDuk tool (parameters ktrim = r, k = 35, mink = 20, hdist = 1, tpe, tbo, qtrim = rl, trimq = 30, minlen = 80) (77). Assembly was performed per sample using SPAdes version 3.15.4 (78) (parameters --meta -m 100t 16k 43,55,77,99,121). Binning was performed using MetaBAT version 2.13 (79), MaxBin version 2.2.6 (80), and CONCOCT version 1.1.0 (81). Contig coverages used in the binning process were generated using Bowtie version 2.3.5 (82) (parameters --phred33 -p 12N 1L 32 --minins 200 –sensitive --maxins 800), mapping each sample read set to their respective assembly. DAS_Tool version 1.1.1 (83) was used to keep the best MAGs per binning tool per sample, resulting in 448 MAGs (Table S2).

### Phylogeny and species delineation

Newly generated MAGs from six *K. sydneyanus* individuals were combined with another 197 generated previously from four individuals (21). A total of 531 reference genomes or MAGs classified as *Alistipes* using the Genome Taxonomy Database (GTDB, 207 release) (62) were downloaded from GenBank (84) (28 October 2022) (Table S2). All genomes were checked for completeness and contamination using CheckM version 1.2.1 (85). Only genomes >75% complete and with <5% contamination were kept. The final data set contained 99 unique good quality *K. sydneyanus-*associated *Alistipes* genomes and 455 associated with other hosts (Table S3).

Pairwise comparisons of all *Alistipes* genomes were undertaken by determining the average nucleotide identities (ANIs) and AFs using anicalculator version 1.0 (63). ANI and AF values were used to generate species-level assignments. In addition, genome clusters sharing >95% and >99% ANI were determined using dRep version 2.3.2 (86). All genomes were retained following dRep and were (re-)classified using the GTDB Toolkit (GTDB-Tk, version 2.1.0) against the 207 database release (62).

The alignment generated by GTDB-Tk, using 120 concatenated gene markers (62), was used to create a phylogenetic tree with FastTree version 2.1.11 (87) with standard parameters, and the output was visualized with iTOL. Additionally, a tree with only *Alistipes* MAGs sourced from the *K. sydneyanus* gut was produced using the GTDB-Tk (61) conserved bacterial marker genes with IQ-TREE version 1.6.12 (parameters -m TEST -b 1000 -nt) (88).

### Genome annotation

Protein-coding genes were inferred using Prodigal version 2.6.3 (parameters -p meta) (89). Annotation of protein coding genes was performed using DIAMOND version 2.0.15 (parameters --max-target -seqs 1 –evalue 0.001) (90) against the SulfAtlas version 2.3.1 (91) and UniProt 2018_09 database release (92). Only matches with >50% coverage and >30% sequence identity with database targets were kept. Protein domains were inferred using HMMER version 3.3.2 (parameters -E 0.001) (93) to search against the PFAM version 32.0 database (94). Orthologous groups were inferred using OrthoFinder version 2.3.3 (95). KOs were annotated using KOFAMscan version 1.3.0 (-e 0.001) (96), keeping the lowest e-value annotation per gene. CAZyme encoding genes were annotated using dbCAN3 version 11 locally using protein mode (66). Only genes annotated with at least two dbCAN tools (HMMER, eCAMI, or DIAMOND) were considered to encode for a CAZyme. Furthermore, dbCAN annotations were harmonized by prioritizing the HMMER annotation, followed by eCAMI.

### Statistical analysis

Correlation coefficients and regression lines were calculated using the stat_cor function from ggpubr (97) and geom_smooth function from the ggplot2 package (97) in R version 4.1.1 (98). A Wilcoxon rank-sum test was performed to identify significant differences in GC content, protein coding density, genome length, and CAZyme density across source groups using the ggpubr (97). *P* values were adjusted using the Benjamin-Hochberg method.

Enriched KEGG categories, KOs, and CAZy families were calculated using Kruskal-Wallis test, followed by a Dunn test using the rstatix (99) package. Results from the Dunn test were corrected using the Bonferroni method.

### Co-localization network

Distinct CAZyme-encoding genes (e.g., different CAZy families and orthogroups) annotated as glycoside hydrolase (GH), polysaccharide lyase (PL), or carbohydrate esterase (CE) within the same CGCs were combined into unique pairs (Table S10). These pairs were searched and counted across all CGCs to estimate how often they are found within gene clusters. Only CGCs containing at least two degradative CAZymes were considered. Networks were visualized using the ggraph package (100).

### Gene expression

RNA reads were trimmed using trimmomatic version 0.39 (101) with adapter removal (trimmomatic parameters HEADCROP:10 SLIDINGWINDOW:4:30 MINLEN:70). Ribosomal RNA reads were removed using SortMeRNA version 2.1 (102). The remaining reads were mapped to the whole dereplicated *K. sydneyanus* community (including non-*Alistipes* representatives) (21) using Bowtie2 version 2.3.5 (82) (parameters: -N 1L 32 --minins 200 –sensitive --maxins 800). Read counts were obtained using the featureCounts tool (parameters: -t exon -g gene_id) (103) and counts (>10 reads per gene). Transcript abundances are shown as transcripts per million (TPM). TPM was calculated per sample as TPM = (number of reads mapped to gene × read length × 1,000,000)/(gene length × *T*), where *T* = ∑(number of reads mapped to gene × read length)/(gene length) (104).

## Data Availability

Metagenomic data were deposited with NCBI under BioProject (PRJNA1029302). Accession numbers for genomes assembled for this study are provided in Table S3.

## SUPPLEMENTAL MATERIAL

Supplemental Information msystems.01007-24-s0001.pdf Supplemental results and Figures S1–S6.

Supplemental Tables msystems.01007-24-s0002.xlsx Tables S1–S12.

ASM does not own the copyrights to Supplemental Material that may be linked to, or accessed through, an article. The authors have granted ASM a non-exclusive, world-wide license to publish the Supplemental Material files. Please contact the corresponding author directly for reuse.
