# Supplementary material for: Hindguts of Kyphosus sydneyanus harbor phylogenetically and genomically distinct Alistipes capable of degrading algal polysaccharides and diazotrophy
Source: mSystems. 2024 Dec 23;10(1):e01007-24. doi: 10.1128/msystems.01007-24 (PMC11748540; doi:10.1128/msystems.01007-24)
Supplement: Supplemental Information — Supplemental results and Figures S1–S6. [file msystems.01007-24-s0001.pdf]

Supplementary Information

Title: Hindguts of *Kyphosus sydneyanus* harbour phylogenetically and genomically distinct *Alistipes* capable of degrading algal polysaccharides and diazotrophy

Authors: Cesar T. Facimoto[a], Kendall D. Clements[a], W. Lindsey White[b], Kim M. Handley[a]

[a]School of Biological Sciences, The University of Auckland, Auckland, New Zealand.

[b]Department of Environmental Science, Auckland University of Technology, Auckland, New Zealand.

Supplementary Results

Of *Alistipes* that were assigned a species designation by GTDB-Tk, ~97% of intra-species pairs shared an AF of >60%. The remaining ~3% of pairs shared AFs below 60% (from 40-60%, Figure S4A), and suggest substantial strain divergence within some *Alistipes* species such as *A. onderdonkii*, *A. putredinis* and *A. senegalensis* (Table S7). Same species AF values below 60% imply considerable strain diversity and phenotypic difference (1, 2). A strong correlation was observed between ANI and AF when comparing all *Alistipes* genome pairs, and a discontinuity was observed between 90-95% ANI (Figure S4C), demarcating 'sequence-discrete' (ecologically differentiated) populations (3, 4). Using >40% AF (which captures most known *Alistipes* species pairs) (Figure S4A) and ≥90% ANI thresholds (genetic discontinuity lower boundary) (3, 4), 89% of pairs displayed ANI >96.5% (Figure S4B) which is the threshold suggested for species delineation (2).

Supplementary Figures

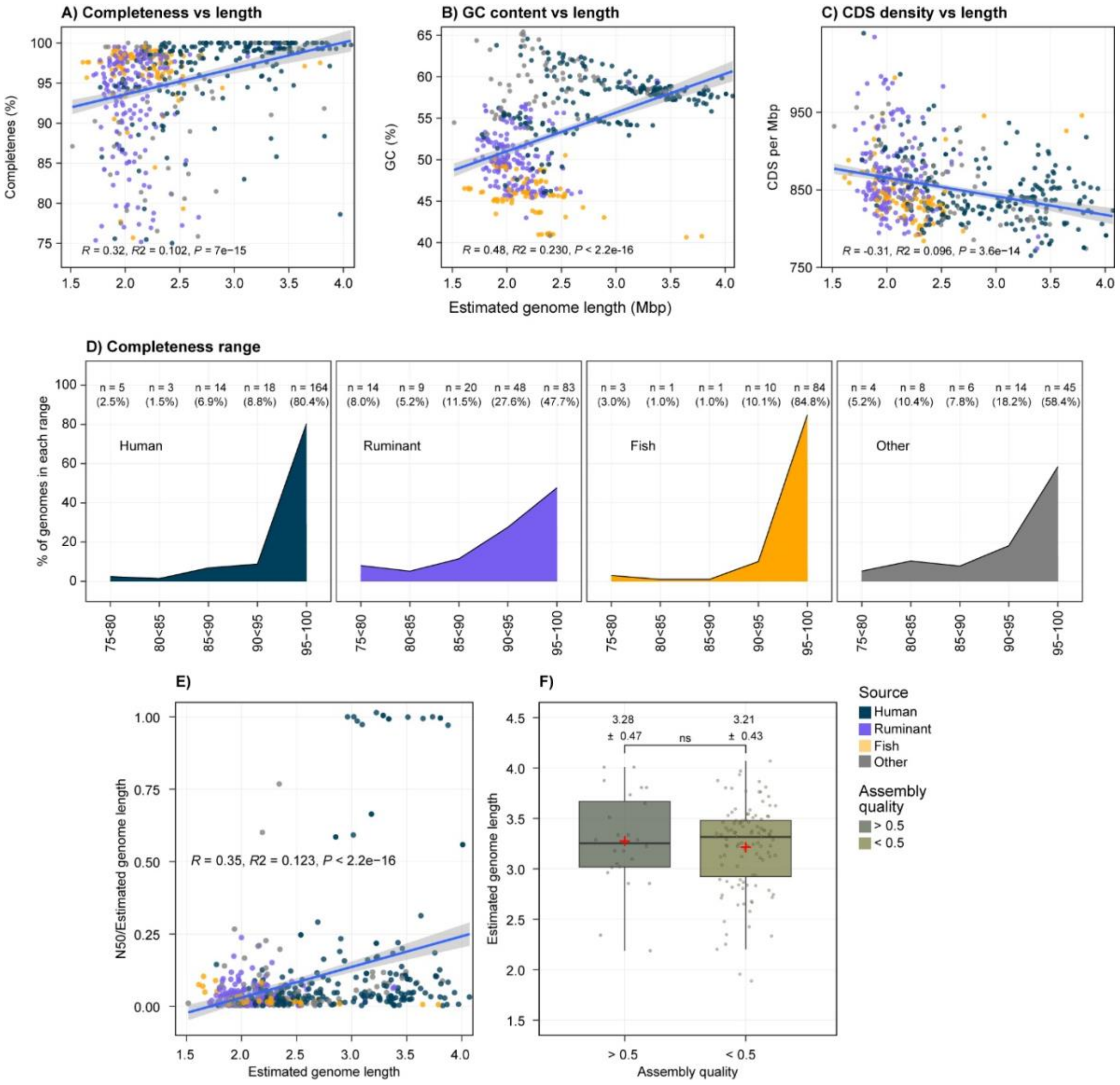

Figure S1 Genomic features and assembly quality. (A-C) Scatter plots show the correlation between completeness, GC content, and protein coding density of genomes compared to estimated genome length. Pearson correlation coefficients and regression lines are shown in the plots. (D) Area plots display the distribution of genomes across completeness ranges. Genome count and their group-wise percentage are shown in the top of the plot. (E) Correlation between the estimated genome length and N50/Estimated genome length ratio. Pearson correlation coefficients and regression lines are shown in the plots. Points are coloured by group and denote the observed values per genome. (F) Estimated genome lengths (corrected for completeness and contamination CheckM estimates) of *Alistipes* species with very high assembly continuity (N50/estimated genome length ratio >0.5) are compared to those with lower assembly continuities (N50/estimated genome length ratio < 0.5) (Table S2). Means (also indicated by red crosses within boxplots) and standard deviations are displayed at the top of the plots.

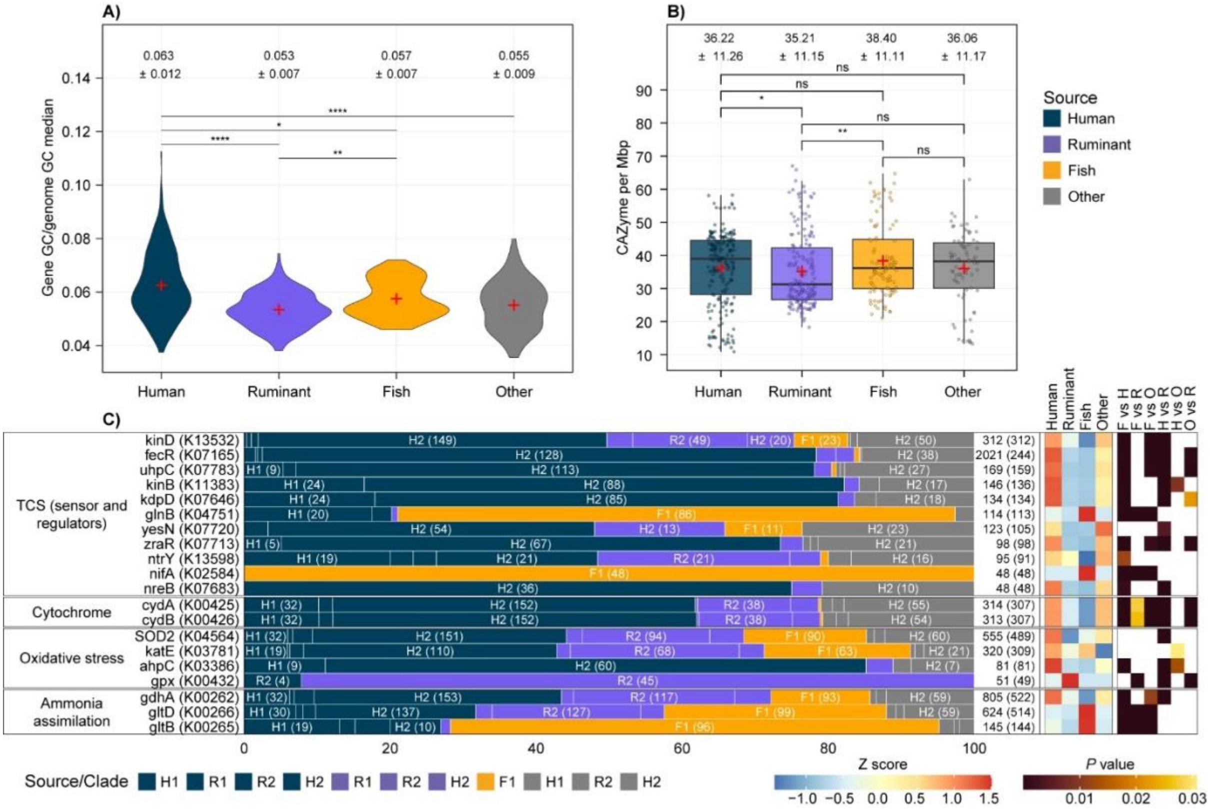

Figure S2 GC content deviation (gene/genome), CAZyme density (CAZyme copies per Mbp), and distribution of environmental adaptation genes. (A) Violin plots displaying the median of gene/genome GC content per genome per source group. Gene deviation (y-axis) was estimated in absolute values by [(gene GC/genome GC)-1]. (B) Boxplot of CAZyme density per source group. Points show the number of CAZyme encoding genes per genome, coloured by source group. The boxes and central line represent the interquartile range and median across genomes per source group, and the whiskers represent the minimum and maximum values within 1.5 times the interquartile range. (A-B) The upper brackets indicate the significance of pairwise groups. Mean values (red cross) and standard deviation values are displayed at the top of the plots. (C) Distribution of genes related to environmental adaptation. Leftmost labels denote the broad functional category of environmental adaptation genes, and gene names and respective KOs are immediately right to the functional category. Bars indicate the percentage of gene copies and their source/clade origin (white outlines) ordered according to the legend at the bottom. White labels denote clade origin and the number of genomes with the gene present. Only source/clades representing >10% of the total copies across all genomes are displayed (white labels). Labels immediately right to bars denote the total number of copies in all genomes and the total number of genomes encoding it. Heatmap immediately right to bar plots represent the mean gene enrichment per source group normalized to z scores. Rightmost heatmap display *P* values (Dunn test with Bonferroni correction, adjusted *P* values <0.05) of pairwise comparisons for each gene across source groups.

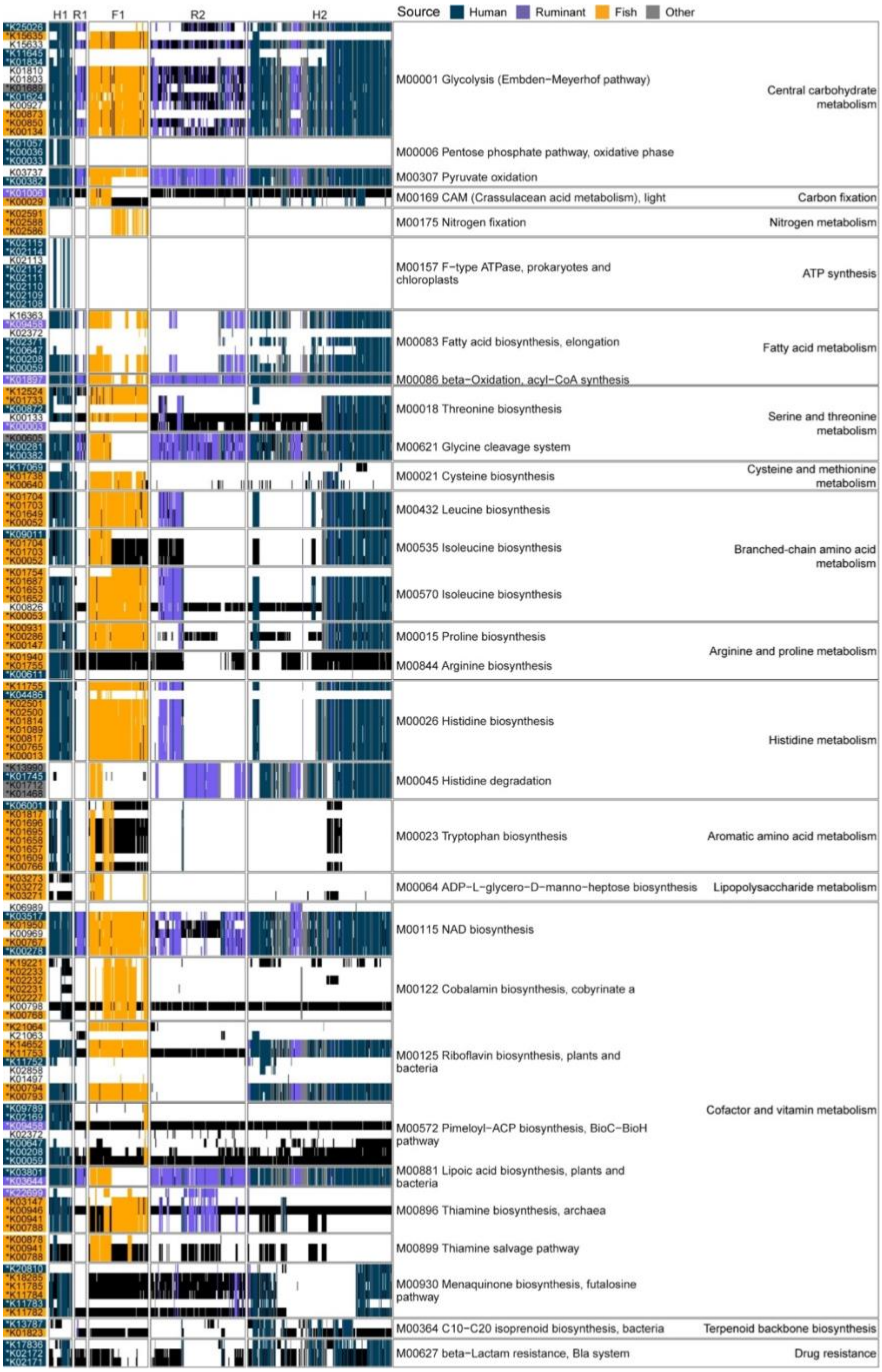

Figure S3 Heat map showing the presence of genes in modules associated with significantly enriched KOs (Dunn test, *P* values <0.05). Modules are displayed if 3/4 of the blocks required for completion in any genome contained significantly enriched KOs. Gene presence is indicated by data points (lines) coloured by source association if the module is 2/3 complete in respective genome, whereas genes present in genomes estimated less than 2/3 complete are coloured black. Absence is indicated by white lines. Each column along the x-axis is a genome. Genomes are ordered by their phylogenetic placement in Figure 1 and breaks separate clades. Predicted KOs are denoted on the y-axis on the left (significantly enriched are marked with an asterisk and coloured by the group comprising their highest mean enrichment), and KEGG modules are shown on the right of the heatmap. Rightmost labels indicate KEGG modules hierarchy. Module completion in each genome was estimated with the ggkegg package (5).

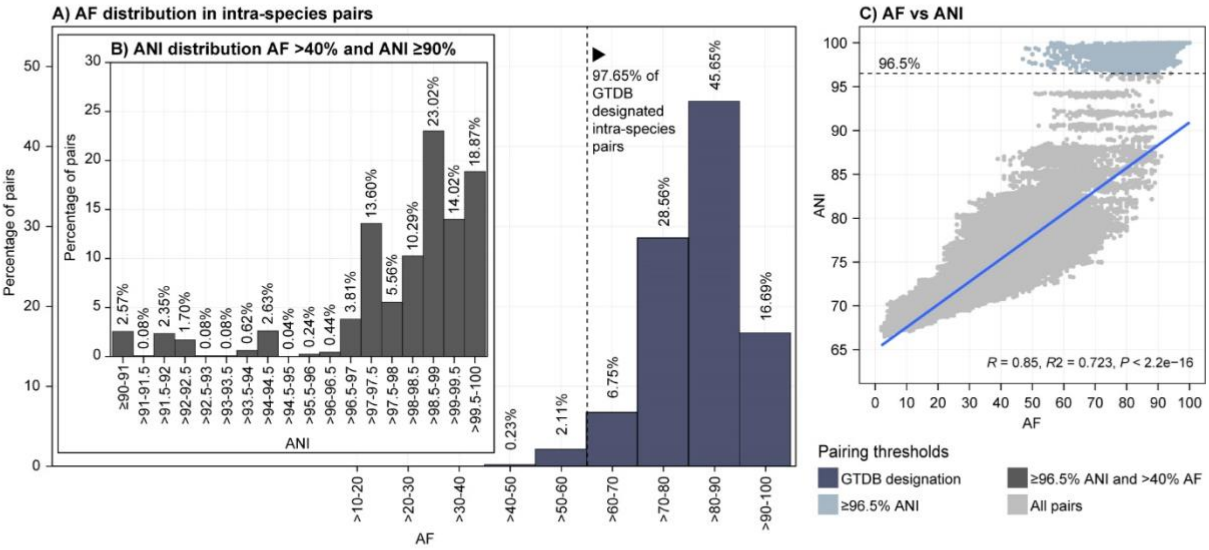

Figure S4 Pairwise alignment fractions (AFs) and genomic average nucleotide identities (gANIs) based on *Alistipes* genomes. (A) Bar plot showing percentage of intra-species genome pairs by alignment fraction. Dark blue bars display the percentage of intra-species pairs where pairs were designated the same species by GTDB-Tk (n = 201 genomes, 3452 pairs). Only species containing at least two genomes were considered. (B) Inset shows the distribution of pairs with >40% AF and ≥90% (dark gray bars) ANI across ANI. (C) Correlation between AF and ANI values across all pairwise genome comparisons (n= 306362 pairs). Points (gray) represent AF and ANI of each pair. Light blue points indicate pairs delineated as same species based on ≥96.5% ANI. Genome pairs were considered same species if exhibited ≥96.5% ANI in at least one of the reciprocal comparisons. Correlation coefficient and significance are displayed within the plot.

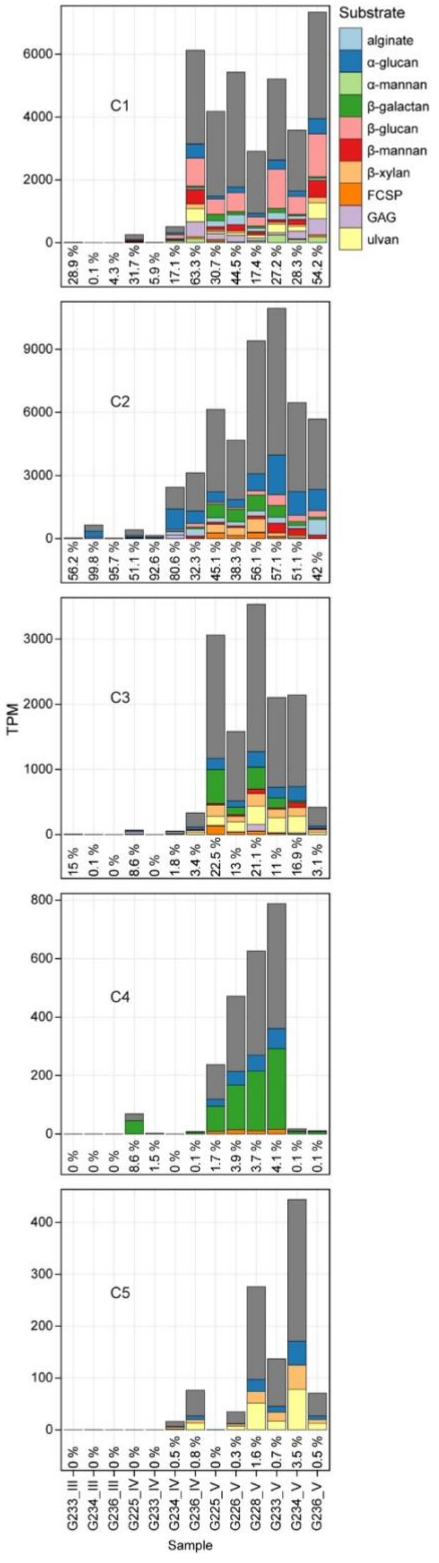

Figure S5 Summed CGC expression per sample across different *Alistipes* clades in the *K. sydneyanus* hindgut. Bars are coloured according to substrate predicted, and unpredicted CGCs are shown in gray. Sample locations along the hindgut are indicated by x-axis labels: III = section III (proximal to the stomach); IV = section IV (the mid hindgut section); and V = section V (the hindgut chamber). Labels at the bottom of bars (within the plot) indicate the relative expression of the clade in each sample (considering the sample total CGC expression). Labels at the middle-left within the plot indicate clade.

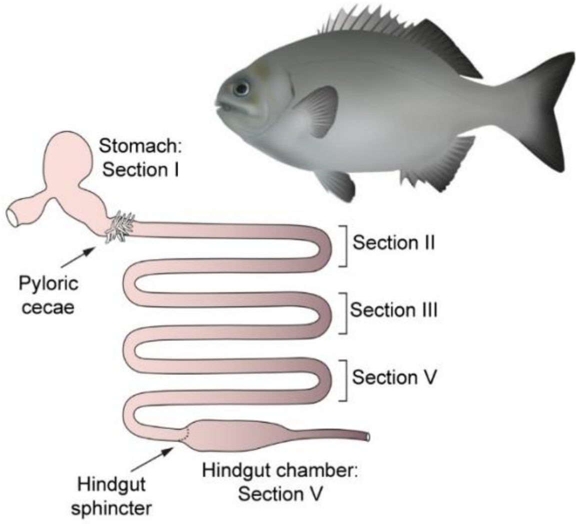

Figure S6 Schematic diagram illustrating the gut anatomy of *Kyphosus sydneyanus*. The stomach is identified as Section I, while the hindgut chamber is designated as Section V. The intermediate intestinal tract is divided into three equal sections, labelled as Sections II, III, and IV.

References

1. Van Rossum T, Ferretti P, Maistrenko OM, Bork P. 2020. Diversity within species: interpreting strains in microbiomes. Nat Rev Microbiol 18:491–506.

2. Varghese NJ, Mukherjee S, Ivanova N, Konstantinidis KT, Mavrommatis K, Kyrpides NC, Pati A. 2015. Microbial species delineation using whole genome sequences. Nucleic Acids Res 43:6761–6771.

3. Konstantinidis KT. 2023. Sequence-discrete species for prokaryotes and other microbes: A historical perspective and pending issues. mLife 2:341–349.

4. Rodriguez-R LM, Conrad RE, Viver T, Feistel DJ, Lindner BG, Venter SN, Orellana LH, Amann R, Rossello-Mora R, Konstantinidis KT. 2024. An ANI gap within bacterial species that advances the definitions of intra-species units. mBio 15:e02696-23.

5. Sato N, Uematsu M, Fujimoto K, Uematsu S, Imoto S. 2023. *ggkegg* : analysis and visualization of KEGG data utilizing the grammar of graphics. Bioinformatics 39:btad622.
